# Mirror exposure following visual body-size adaptation does not affect own body image

**DOI:** 10.1098/rsos.221589

**Published:** 2023-08-16

**Authors:** Thomas Chazelle, Michel Guerraz, Richard Palluel-Germain

**Affiliations:** Univ. Grenoble Alpes, Univ. Savoie Mont Blanc, CNRS, LPNC, 38000 Grenoble, France

**Keywords:** visual adaptation, body representation, body image distortion, body size and shape misperception, self-specific effects, media influence

## Abstract

Prolonged visual exposure to large bodies produces a thinning aftereffect on subsequently seen bodies, and vice versa. This visual adaptation effect could contribute to the link between media exposure and body shape misperception. Indeed, people exposed to thin bodies in the media, who experience fattening aftereffects, may internalize the distorted image of their body they see in the mirror. This preregistered study tested this internalization hypothesis by exposing 196 young women to an obese adaptor before showing them their reflection in the mirror, or to a control condition. Then, we used a psychophysical task to measure the effects of this procedure on perceptual judgements about their own body size, relative to another body and to the control mirror exposure condition. We found moderate evidence against the hypothesized self-specific effects of mirror exposure on perceptual judgements. Our work strengthens the idea that body size adaptation affects the perception of test stimuli rather than the participants' own body image. We discuss recent studies which may provide an alternative framework to study media-related distortions of perceptual body image.

## 1. Introduction

Media exposure has long been identified as a risk factor for body image distortion [1]. Specifically, mere exposure to thin bodies has been associated with overestimation of one's own body size [2–4]. This media influence could pose a public health problem, as misperception of one's own body shape is a risk and severity factor in eating disorders such as anorexia nervosa [5]. This misperception resembles the aftereffects of visual adaptation to extreme body sizes [6–10]. In this study, we investigated the hypothesis that adaptation can specifically affect participants' own body image.

In classical visual adaptation effects, exposure to a given feature leads to a selective perceptual aftereffect in the direction opposite to the adapted feature [11]. For example, in the waterfall illusion, prolonged exposure to the downward movement of a waterfall causes an illusory aftereffect of upward movement [11]. In the body-size visual adaptation paradigm, the adapted feature is often adiposity: participants are exposed to a picture of a large or thin body for a few minutes [6–8,12]. Before and after this adaptation phase, participants have to judge whether a displayed test body is thinner or larger than a standard stimulus, typically themselves. The size of the test body is varied across trials, so that participants' responses can be fitted to a psychometric curve. This allows the computation of a point of subjective equality (PSE), that is, the body size to which participants should randomly respond when asked to choose whether it is thinner or fatter than the standard stimulus. At the PSE, researchers infer that the test stimulus and the standard stimulus are judged as being the same size. Several studies used this type of procedure and showed that adaptation to large bodies produces a thinning aftereffect on following stimuli, whereas the opposite fattening aftereffect is found after exposure to thin bodies [13].

Adaptation provides a neurocognitive framework that makes experimentally testable assumptions about the aftereffects of prolonged exposure to bodies. From this perspective, adaptation aftereffects may explain through visual mechanisms how women's body image can get altered by exposure to thin bodies in the media [2]. Authors have indeed proposed that visual adaptation could help develop an ‘experimental model of body image distortion’ [8, p. 9]. Some authors have taken this idea a step further, interpreting adaptation aftereffects as proof that ‘[visual adaptation] impacts the internal representation of body size' [10, p. 9]. The perceptual component of body image, understood as a stored representation [13] with which ‘a person can judge the physical dimensions of their own body' [14, p. 35], could well be influenced by visual adaptation. This is made possible by the fact that adaptation can produce aftereffects on stimuli of different identities, although they may be weaker than the effects for same-identity stimuli [8,12]. This is a crucial result, as adaptation to the thin bodies of celebrities and media figures can then be thought to influence people's own body image [4].

However, the interpretation of visual adaptation aftereffects must be cautious, as it can suffer from the ‘El-Greco fallacy' [15]. The term derives from the misinterpretation of the elongated shapes in El Greco's paintings as the result of his astigmatism. Firestone & Scholl pointed out that if astigmatism had distorted his vision, he should have seen his canvas as similarly distorted, thus nullifying the distortion to outside observers. In the context of body-size adaptation aftereffects, if adaptation affects own body image similarly to the test stimuli, all bodies should be perceived as larger (or thinner), resulting in the absence of any detectable aftereffect in a visual judgement task. Consequently, the fact that these aftereffects have been observed after prolonged exposure to an extreme body size suggests that adaptation only affects test stimuli, whereas participants' internal body image remains unchanged, contrary to some interpretations of adaptation aftereffects (e.g. [10]). Ambroziak *et al*. [16] reached a similar conclusion, noting that ‘if adaptation affects all bodies equally, the relative difference between one's own body and other bodies should not change' (p. 3). In three experiments, they replicated the adaptation effect while varying the standard stimulus (to which test stimuli are compared) following adaptation. If the participants' judgements were altered when judging the size of their own body relative to that of another person's, this would be a good reason to think that the participants' own body image is indeed specifically distorted following adaptation. On the other hand, if only the perception of test stimuli is affected by adaptation, the same biases should arise regardless of which body the test bodies are compared to. Indeed, Ambroziak *et al*. did not detect any difference in body-size adaptation aftereffects when they asked whether the test stimulus was fatter than the participant themself or than any other reference body (an average body, the experimenter's body or a celebrity's body). By itself, body-size adaptation does not seem to change the participants' body image. The authors concluded that it is more parsimonious, at this stage, to say that it only affects the perception of test stimuli (see also [17] for a similar conclusion). However, the possibility that adaptation aftereffects alter people's daily visual experience of their own bodies is not addressed in these studies. Indeed, after visual adaptation, ‘the perception of subsequently seen bodies, including that of the mirror-gazing consumer, may be biased such that they are seen as larger […] than they really are, with unusually thin […] figures being rated as normal' [4, p. 4]. That way, the distorted perception that people have of their own body while experiencing adaptation aftereffects could actualize and distort their body image, in a process referred to as internalization [4]. In other words, the adaptation aftereffect altering body perception in general could coexist with self-specific effects due to internalization, and the perceptual body image may remain distorted when adaptation later fades out. By self-specific effects, we mean effects that arise only or differently when judging one's own body but not the body of others. If this hypothesis is correct, there should be self-specific effects when participants have been exposed to their own body after adaptation. ‘For example, if an individual perused “thinspiration” images on social media before observing him/herself in a mirror or photograph, they would be likely to perceive their body to be larger than it is. This enlarged percept may then be used to update the stored representation of their own body' [13, p. 3]. In fact, mirror exposure was mentioned in several studies as a behaviour by which people might internalize a distorted image of themselves [4,9,13,18].

In this study, we tested this internalization hypothesis by investigating whether exposure to one's own body following adaptation to a body with a high body mass index (BMI) could produce self-specific effects on visual judgements. Following the internalization hypothesis, participants should represent their body as thinner after exposure to the perceptually thinned reflection of themselves they see after adaptation to a large body [4,13]. Then, when comparing pictures of bodies to their own body, the cumulative influences of the adaptation aftereffect and of the internalization effect should lead to a smaller difference between perceptually thinned test stimuli and participants' own thinned body image. Participants should therefore exhibit reduced adaptation aftereffects when comparing test stimuli with their own body, whereas the detected aftereffects should be greater when comparing test stimuli with another person's body and in absence of mirror exposure following adaptation.

Finding this interaction effect between time (before versus after adaptation), target (judging one's own body versus another person's) and exposure (mirror exposure after adaptation versus a control condition) would support the internalization hypothesis. Failure to identify such an effect would strengthen the remarks made by Ambroziak *et al*. [16], indicating that adaptation aftereffects observed in the laboratory are primarily explained by misperception of the test stimuli.

## 2. Material and methods

### 2.1. Power analysis

We used the Superpower R package [19] to conduct an *a priori* power analysis on our ANOVA design. This package allows simple estimation of the effect size by setting group means and standard deviation. *Α* was set at 0.05 and power at 1 − *β* = 0.90. We made assumptions about the results based on previous work using the same stimuli and similar procedures, namely, the three studies of Ambroziak *et al*. [16] and our own unpublished replication of the adaptation effect, available in the electronic supplementary material. Further details and justifications are available on the preregistration form of our study [20]. We notably hypothesized that self-specific underestimation when adaptation is followed by mirror exposure should lead to a drop of a third of the PSE change (when compared with control conditions). The hypothesized data correspond to $\eta_{p}^{2}=0.03,$ that is, a small-to-medium interaction effect [21]. With these settings and 10% participants as a safeguard, we estimated we would need to recruit 374 participants in total (187 per mirror exposure group) to detect the interaction effect predicted by the internalization hypothesis. However, this preregistered power analysis underestimated the recruitment constraints. After failing to recruit more than 213 participants, we performed another power analysis on the basis of the upper bound of the 95% confidence interval for the observed effect size of the interaction effect, that is, $\eta_{p}^{2}=0.014$ (see Results). We found that 540 participants would then be needed to detect such a small effect at *α* = 0.05 and 1 − *β* = 0.80. In the light of these results that rely on the highest estimate of the effect size, we reasoned that this sample size was not reachable, especially in the context of a laboratory study, and that resuming data collection would waste resources.

### 2.2. Participants

This study included 196 participants aged between 18 and 27 years old (*M* = 19.56, s.d. = 1.67), with BMIs ranging from 17.5 to 33.1 kg m^−2^ (*M* = 21.44, s.d. = 2.96) computed from their self-reported height and weight. We initially recruited 213 French women through an online post and rewarded them with course credit. After the study was completed, we excluded six participants who had a BMI under 17.5 kg m^−2^. This was a preregistered exclusion criterion, alongside having a BMI over 38.8 kg m^−2^, corresponding to the 5% lowest and highest BMIs for French women under the age of 29 [22]. We further removed three participants due to missing data and eight participants due to undetected answer key inversions which resulted in flawed data (see Results). Participants were all women due to the nature of the stimuli. We did not control for their skin colour. They had normal or corrected-to-normal vision and declared no history of eating disorder or body dysmorphic disorder. All participants gave the above information and their consent in a form prior to the experiment. The experiment was approved by the Grenoble Alps Research Ethics Committee.

### 2.3. Materials

During baseline, adaptation, post-test and deadaptation phases, participants sat at about 40 cm from a computer screen (15.6 inches, refresh rate: 30 Hz, resolution: 1920 × 1080), whereas during mirror exposure and waiting phases, they stood in front of a standing mirror. The mirror was 165 cm × 47 cm with a 10° inclination. Participants stood on two marks on the ground, 150 cm and 160 cm away from the mirror and 15 cm away from each other, such that participants faced the mirror with their body at an angle of approximately 45°. The mirror was covered by a black sheet that was only removed during the mirror exposure phases.

For the assessment of body size perception, we used the same image set as Ambroziak *et al*. [16]. It consists of 89 images of a white woman in underwear, oriented laterally by about 45°, with a BMI ranging from 13 to 35 kg m^−2^ by steps of 0.25. These stimuli are available online [23]. Participants had to determine whether the body displayed on screen was thinner or fatter than a standard stimulus, that is, a reference body. This reference was either their own body (self condition) or the body of Emma Watson (other condition). Prior to the experiment, we ensured that all participants were familiar with Emma Watson's appearance by asking them whether they knew her and how she looks.

All participants underwent adaptation to a high-BMI body. We made this choice to increase statistical power, avoid a multiplication of the number of experimental conditions, of the sample size, and to limit the duration of the experiment. We chose large adaptors as some people, such as women with eating disorders, are less sensitive to adaptation to low-BMI bodies, possibly because of media ‘preadaptation' to thin bodies [24], even though this choice was somewhat arbitrary considering that women with eating disorders were excluded. The large adaptor consisted of the maximum BMI of the picture set (35 kg m^−2^).

### 2.4. Procedure

**Figure 1.**
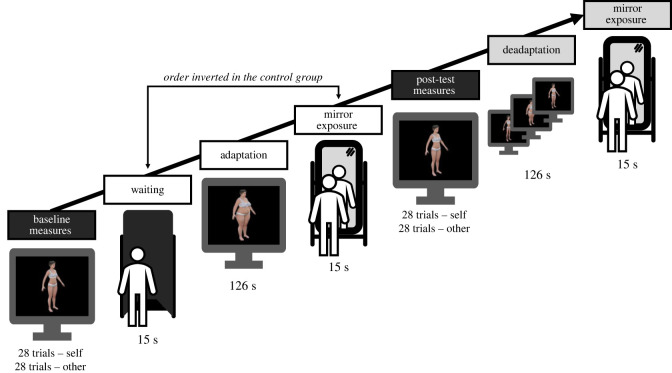
Overview of the procedure. Black boxes correspond to the measures, white boxes to the adaptation–exposure induction and grey boxes to the deadaptation phase included for ethical reasons. Participants were randomly assigned to either late mirror exposure (depicted here) or early mirror exposure (the control condition, in which mirror exposure and waiting phases were inverted). Within measure blocks, self and other conditions were counterbalanced across participants in ABAB order.

The procedure is outlined in figure 1. Body size perception was assessed twice, in distinct baseline (pre-adaptation) and post-test (post-adaptation) blocks. After the baseline assessment, participants went through an adaptation phase, that is, they had to look at the large adaptor. There were two mirror exposure conditions, ‘late' mirror exposure and control ‘early' mirror exposure. In the ‘late' mirror exposure condition, participants looked at their reflection right after the adaptation phase. In the control condition, rather than no exposure at all, mirror exposure took place right before the adaptation phase (‘early' mirror exposure). We made this choice because brief mirror exposure has been linked with psychological changes such as objective awareness, self-evaluation and negative affect [25]. Brief mirror exposure can lead to a temporary increase of body dissatisfaction [26], which seems to modulate visual adaptation effects [24]. Mirror exposure also provides visual feedback that could enhance the precision of body size estimation [27]. By contrasting the effect of mirror exposure before and after adaptation, we tried to isolate the specific perceptual effect expected according to the internalization hypothesis. For ethical purposes, the post-test measurements were followed by a deadaptation phase and a final exposure to the mirror.

To assess participants' body size perception, we used a psychophysical procedure adapted from the one of Ambroziak *et al*. [16] with an implementation of QUEST on Matlab's Psychtoolbox, available in the electronic supplementary material. QUEST is an adaptive psychometric method used to estimate psychophysical response curves and thresholds with fewer trials than other non-adaptive methods [28]. Unlike Ambroziak *et al*. we performed one staircase by condition, initialized at 25.25 kg m^−2^. This corresponds to the mean BMI in a previous sample of young French female psychology students from an unpublished replication, added to the weighted average of overestimation in baseline trials in Ambroziak *et al*.'s three studies and our own replication (+3.6 kg m^−2^). Each staircase consisted of 28 trials instead of the 32 trials we initially preregistered [20], because 28 trials were sufficient to reach a stable PSE in pretests. Participants' responses allowed QUEST to choose the BMI of the next stimulus. Once the 28 trials had been performed, the PSE was obtained. PSEs were computed using QUEST and corresponded to the estimated BMI for which participants should answer that the test image is thinner (or fatter) than the reference in 50% of trials. The PSE can be understood as a proxy for which body participants think has a BMI closest to the reference they are judged against. Each participant completed four staircases, corresponding to baseline and post-test assessments in both self and other conditions. Self and other conditions were counterbalanced across participants in ABAB order.

In baseline trials, participants judged whether the displayed body was thinner (left arrow of the keyboard) or fatter (right arrow of the keyboard) than a reference (i.e. the target: self or other, in two different blocks). Each body was displayed for 1 s following a fixation cross (500 ms). The reference body was not displayed on the screen; that way, participants had to compare the test body to a stored reference in both conditions [16]. The screen remained grey until a response was obtained. During the first 10 trials of the first staircase, the experimenter remained standing behind the participant and monitored the use of the correct response keys. The experimenter reset the task and explained it again if the response keys were manifestly reversed.

During the adaptation phase, participants looked at the high-BMI adaptor for 120 s. As in other studies [16], to maintain participants' attention, the adaptation stimulus flashed for 200 ms every 4000 ms. The adaptation phase lasted 126 s with these flashes included.

In post-test trials, participants performed the same task as during the baseline trials. However, before each trial, the adaptation stimulus was presented again for 4000 ms in order to maintain visual adaptation throughout the task (‘top-up adaptation' [8,16]). Participants were asked to look at all displayed images but to perform the task only on the second body.

Participants were exposed to the mirror before or after adaptation, depending on their experimental group. A waiting phase was added so that the same time elapsed between measures and adaptation regardless of the condition (figure 1). Mirror exposure and waiting phases started with the participant standing in front of a mirror covered by a black sheet, eyes closed. In the waiting phase, the experimenter asked the participant to start looking at the black sheet at the prompt. In the mirror exposure phase, the experimenter lifted the black sheet and asked the participant to start looking at the reflection of her body, and not her face, at the prompt. In both cases, the experimenter then gave the signal to look and started a 15 s timer. When the timer stopped, the experimenter covered the mirror again while asking the participant to go back to the computer. During waiting and mirror exposure phases, the experimenter was standing outside the participant's visual field. Third-party timing during pilots established that the effective duration of the exposure varied between 15 and 18 s.

For ethical purposes, we also included a deadaptation phase at the end of the experimental protocol. Participants looked at bodies with BMIs ranging from 13 to 33 kg m^−2^ by steps of 1 in random order. Each body was displayed for 6 s, for a total of 126 s of deadaptation. Afterwards, to prevent any potentially long-lasting perceptual effects of our protocol, participants looked at their reflection in the mirror for another 15 s. They were then debriefed about the purpose of the study and given the contact of the study supervisor in case they experienced any discomfort or unusual experience after the study. No participant reported such long-lasting side effects.

The psychophysical procedure we used implies that changes in PSE can unveil the changes in body size perception we are interested in. The body-size visual adaptation literature indicates that participants should see test images as thinner after adaptation to the high-BMI stimulus. For a same-sized body, there should be more ‘the test body is thinner' responses after adaptation than before. This change should be captured by a main effect of time (baseline versus post-test) on the PSE, which would then be higher in the post-test block than in the baseline block [4,9], regardless of the target reference stimulus [16]. If the internalization hypothesis is correct, participants should also internalize a thinned version of themselves when exposed to the mirror after adaptation, reducing the difference with the thinned test stimuli [4,13]. Therefore, there should be a lesser difference of PSE between baseline and post-test in the self condition than in the other condition. If this difference of PSE changes is due to adaptation aftereffects and not to non-specific effects of mirror exposure, it should not emerge in the control early exposure condition, in which mirror exposure takes place before adaptation. This is why we hypothesize a double interaction between time, target reference stimulus, and mirror exposure condition.

## 3. Results

Dataset formatting, outlier inspection, and plots were performed on R 4.0.5 [29]. Inferential analyses were run on JASP 0.16.2 [30]. The dataset is openly available in the electronic supplementary material. Statistical outliers were screened for each analysis using preregistered rules [20]. Preregistered analyses are presented in the confirmatory section of the results, whereas unplanned analyses are presented in the exploratory section. Notably, we performed exploratory Bayesian analyses to assess support for the null hypothesis. In confirmatory frequentist analyses, we used a cut-off for significance of *α* = 0.05. In exploratory frequentist analyses, *p*-values are reported for information purposes. In Bayesian analyses, *BF_01_* > 150 was interpreted as very strong evidence for the null, *BF_01_* > 20 as strong evidence for the null, and *BF_01_* > 3 as positive evidence for the null [31].

### 3.1. Confirmatory analyses

**Figure 2.**
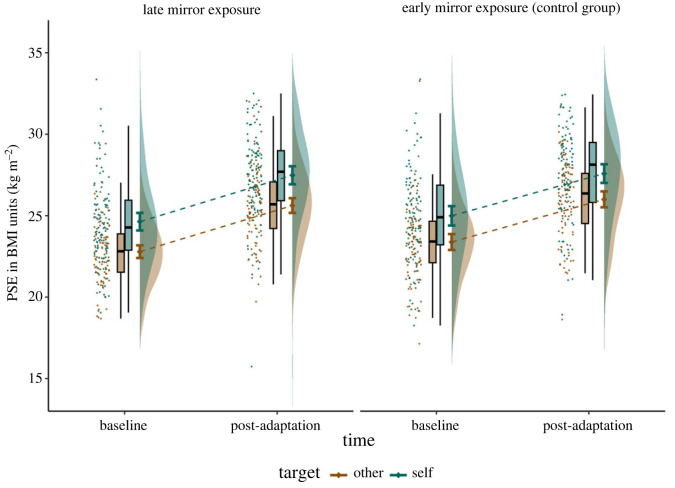
PSE by time (baseline versus post-adaptation), target (self versus other) and exposure group (late mirror exposure versus early exposure control). In each condition, the raincloud plot depicts the individual data, box plots, group means with 95% confidence intervals and violin plots (from left to right).

**Figure 3.**
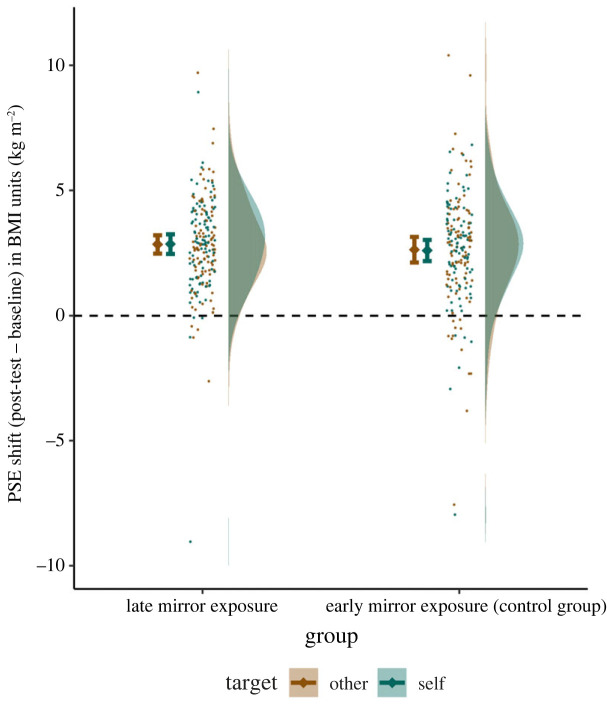
PSE shift (post-test minus baseline) by target (self versus other) and exposure group (late mirror exposure versus early exposure control). The dashed line at 0 shows the absence of difference between baseline and post-test. Values above 0 correspond to the expected adaptation aftereffect, with increased PSEs in post-test compared to baseline. Error bars denote 95% confidence intervals.

For each experimental condition, QUEST computed a PSE corresponding to the mean of the estimated distribution, leading to four PSEs per participant (baseline and post-test in both self and other conditions; figure 2). Of the 207 participants included in the study, we removed 11 participants due to missing data (three participants) or undetected answer key inversions which resulted in minimum or maximum obtainable PSE values, because of the adaptive nature of QUEST (eight participants). All analyses were therefore performed on 196 participants. To better visualize the adaptation aftereffect, PSE shifts (post-test PSE minus baseline PSE) in self and other conditions in both mirror exposure groups are plotted in figure 3. On average, when comparing test stimuli to oneself (self condition), PSEs increased from 24.81 (s.d. = 2.80) to 27.53 (s.d. = 2.78), with shifts ranging from −9.04 to +8.93, *M* = +2.72, s.d. = 2.02, *t*_195_ = 18.8, *p* < 0.001, Cohen's *d_z_* = 1.35. When test stimuli were compared to Emma Watson (other condition), PSEs increased from 23.08 (s.d. = 2.19) to 25.81 (s.d. = 2.33), with shifts ranging from −7.56 to +10.40, *M* = +2.73, s.d. = 2.19, *t*_195_ = 17.5, *p* < 0.001, Cohen's *d_z_* = 1.25.

We performed a 2 × 2 × 2 mixed ANOVA on PSEs with time (baseline versus post-test) and target reference stimulus (self versus other) as within-participants factors and mirror exposure group (late exposure versus control early exposure) as a between-participants factor. We replicated previous findings regarding the adaptation aftereffect, as indicated by a strong effect of time on PSEs, *F*_1,194_ = 474.3, *p* < 0.001, $\eta_{p}^{2}=0.710$. After viewing the image of a woman with a high BMI, the BMI value for which participants should respond randomly increased by 2.73 (s.d. = 2.11) on average across all conditions (figures 2 and 3). This can correspond to perceiving test stimuli as thinner, the reference as larger, comparing them differently, or any combination of these explanations. Unsurprisingly, there was also a main effect of target, *F*_1,194_ = 105.3, *p* < 0.001, $\eta_{p}^{2}=0.352$. Participants judged Emma Watson as thinner (*M* = 24.44, s.d. = 2.64) than themselves (*M* = 26.17, s.d. = 3.10). This 1.7 points increase is consistent with the fact that participants' actual BMI was higher on average (*M* = 21.4) than that of the actress (around 19). On the other hand, the predicted time × target × exposure double interaction effect was not significant, *F*_1,194_ = 0.02, *p* = 0.90, $\eta_{p}^{2}=0.001,$ 95% CI on the effect size: [0, 0.014] (figures 2 and 3). No other effect was significant or exceeded $\eta_{p}^{2}=0.01$.

### 3.2. Exploratory analyses

**Figure 4.**
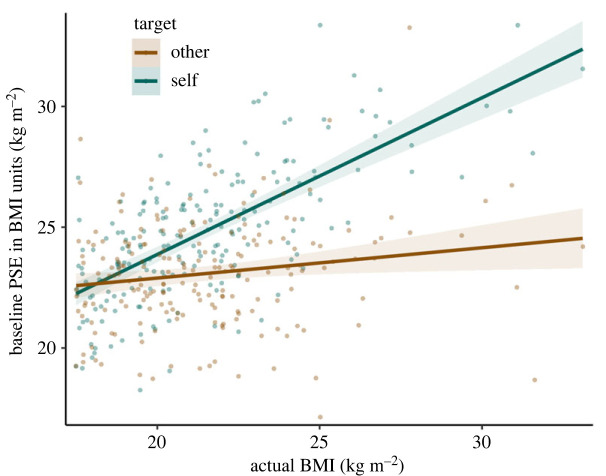
Baseline PSE by participants' actual BMI and by target condition. Error bands denote 95% confidence intervals.

Participants' self-reported BMI correlated with baseline PSEs in the self condition, *r*_194_ = 0.68, 95% CI [0.60, 0.75], *p* < 0.001, indicating that participants performed the task appropriately: QUEST's estimates of the threshold strongly correlated with participants' actual body shape, when asked about themselves (figure 4). There was also a much smaller correlation between participants' BMI and baseline PSEs in the other condition, *r*_194_ = 0.17, 95% CI [0.03, 0.30], *p* = 0.02 (figure 4). This result should be taken with caution, as this correlation is weak [21], results from an exploratory analysis became weaker when an outlier was removed, and contradicts previous findings about the impact of women's own BMI on their estimates of others' body size [16,32,33].

We also ran an exploratory mixed model analysis including time, target, exposure, BMI, and their interactions as predictors of PSE, and time and target as random factors. We estimated *p*-values with Satterthwaite's method in lme4 [34] and lmerTest [35]. There were a total of 16 predictors, so we considered the effect as noteworthy when *p* < 0.05/16 ≈ 0.003. Again, we found a main effect of time, *t*_192_ = 206.6, *p* < 0.001, $\eta_{p}^{2}=0.728,$ and target, *t*_192_ = 21.9, *p* < 0.001, $\eta_{p}^{2}=0.523$. There was a main effect of BMI, *t*_192_ = 8.1, *p* < 0.001, $\eta_{p}^{2}=0.267,$ and an interaction effect between BMI and target, *t*_192_ = 12.8, *p* < 0.001, $\eta_{p}^{2}=0.477$. This interaction trivially corresponds to a greater effect of participants' BMI on the PSE when the target of the comparison was oneself rather than Emma Watson. BMI was a significant predictor of PSE in the self condition, *t*_221.7_ = 13.8, *p* < 0.001, $\eta_{p}^{2}=0.514,$ but not in the other condition, *t*_223.6_ = 1.56, *p* = 0.12, $\eta_{p}^{2}=0.013$.

Finally, to quantify the level of evidence against the hypothesized double interaction, we conducted 2 × 2 × 2 Bayesian mixed ANOVA with the same factors and computed inclusion and exclusion Bayes factors against matched models [36]. In line with frequentist analyses, there was strong evidence in favour of the inclusion in the model of both time, log(*BF*_incl_) = 159, and target, log(*BF*_incl_) = 73. There was also moderate evidence against the inclusion of the time × target × exposure double interaction as a relevant factor, *BF*_excl_ = 5.71. An exploratory comparison of models with every combination of these fixed effects identified a model including main effects of time and target as the best supported by the data against the null, log(*BF*_10_) = 203.

## 4. Discussion

Exposure to low-BMI bodies in the mass media is linked to body shape misperception, in association with body dissatisfaction and eating disorders [1]. Some authors have mentioned [4,13,18] or argued [10] that this phenomenon is partly due to adaptation aftereffects caused by prolonged and repeated exposure to low-BMI bodies. After adapting to thin media figures, people should see all bodies as larger than before and, according to the internalization hypothesis, the distorted reflection of their own body in the mirror could influence their perceptual body image [13]. Our study provides evidence against this internalization hypothesis. Although we replicated the typical adaptation aftereffect, mirror exposure following adaptation did not induce any detectable self-specific perceptual effect, compared to control exposure and target conditions. This result suggests that body-size adaptation, even when followed by mirror exposure, produces aftereffects due to distortions that target the perception of test stimuli, rather than participants' own perceptual body image.

Our result is coherent with the conclusions reached by Ambroziak *et al*. [16], who failed to find any specific alteration of perceptual body image in the same forced choice task. Similarly, Bould *et al*. [17] found no own-body overestimation after repeated exposure to thin bodies during 5 min, twice a day, for a week. On the contrary, participants responded to this procedure as if they underestimated their own size (or overestimated the size of test stimuli). This result is coherent with an adaptation aftereffect confined to seen test stimuli: if adaptation to thin bodies leads to overestimation of bodies they see, participants should perceive the test stimuli as larger, and thus select a thinner-than-normal stimulus as one that most resembles their unaltered body image. Using different measures, Zopf *et al*. [18] also failed to detect adaptation-induced body image distortions with a tactile distance estimation task. In their study, they measured the aftereffects of visual adaptation on both a classic visual judgement task and a tactile distance estimation task in which participants matched a calliper to the distance between two tactile stimuli on their abdomen. They replicated the adaptation aftereffect on visual judgements, but obtained no evidence that tactile distance estimations could be influenced by adaptation. The authors concluded that visual adaptation aftereffects do not transfer to multisensory representations of the body. This interpretation echoes the debates surrounding the number of body representations [37,38], as it can be interpreted as evidence that visual adaptation can influence a visual body representation whereas it does not impact the distinct somatosensory body representation underlying the tactile distance estimation task. This result can also be interpreted as further evidence that body-size adaptation by itself does not influence participants' representation of their own body. In line with the internalization hypothesis [4,13], Zopf *et al*. underline that adaptation aftereffects could have influenced somatosensory tasks if ‘the visually adapted individual [had viewed] their own body (e.g. in a mirror) while visually adapted to extreme body types' [18, p. 12].

Our results weaken this mirror argument, according to which mirror exposure after adaptation could account for the internalization of an adaptation-distorted reflection in the perceptual body image. We provide moderate evidence that mirror exposure following body-size adaptation does not produce notable self-specific effects, strengthening the criticism first issued in other studies [16,17]. Considering our results, it appears that body-size adaptation only influences the perception of test stimuli, and this bias is not internalized in body image following a brief mirror exposure. In the absence of self-specific effects and without additional evidence for the internalization of adaptation aftereffects, adaptation does not explain how body image distortions can emerge following exposure to extreme body shapes. Nevertheless, this conclusion must be considered in the light of the limitations of our study. Due to recruitment constraints that we underestimated in the *a priori* power analysis, we were unable to reach the sample size initially planned [20]. However, it should be noted that the null result obtained in our sample is not inconclusive: a Bayesian analysis indicated support for a model that does not include the interaction of interest, *BF*_excl_ = 5.71, exceeding the commonly used threshold of *BF*_01_ = 3 [31]. Our final sample size (*N* = 196) is also larger than that typically encountered in body-size adaptation research. In the spirit of a sequential analysis, we also conducted a second power analysis using the highest estimate of the effect size we observed. We found that 540 participants would then have been needed to achieve adequate power, a number we consider unrealistic for a laboratory study even under these favourable conditions (see Power analysis). Therefore, we argue that the data we have gathered against the existence of self-specific mirror effects, although not decisive, add to the existing evidence against the internalization hypothesis.

The stimuli we used induce limitations to our conclusions. First, the use of the same stimuli as adapters and as test stimuli might cause low-level effects. However, this low-level adaptation can cohabit with a higher-level adaptation, related to adiposity [39]. As its effect is diminished by low-level variations [39], the effect is likely to be overestimated if anything, so this limitation is not critical. Second, the fact that the stimuli are computer-generated images and not photographs may be problematic if the effects are different with real bodies, as is the case in some studies [40]. Third, these stimuli contain a face, which easily attracts attention and may itself be subject to effects known as face aftereffects [41]. This limitation should be kept in mind despite the use of a fixation point pointing to the body centre of the stimuli in our study. Furthermore, the same idiosyncrasies can be feared with regard to the standard stimulus used (the actress Emma Watson). Specifically, while she had the advantage of being known to all our participants, this actress is also known for her roles in the Harry Potter films, which could increase the variability of this reference across participants. As we are interested in the adaptation effect rather than the absolute value associated with the standard stimulus, this limitation is of little theoretical concern here. The absence of a low-BMI adaptor condition is also a limitation, as it does not allow us to check whether the results are symmetrical in both adaptation directions. Future studies should seek to replicate our results with different adaptors, test stimuli and standard stimuli, to avoid our results being driven by idiosyncrasies of our particular stimuli. The short duration of mirror exposure (15 to 18 s) in the present study and the fact that there was only one exposure phase could explain the lack of effect. According to the internalization hypothesis, participants could update their body image with the distorted perception of themselves in the mirror after adaptation [13]. Rather than a single, brief mirror exposure, people typically engage in numerous, longer mirror-gazing sessions in their daily lives [42]. These prolonged and repeated exposures could, for their part, update body image after adaptation by thin media figures, whereas a single and brief exposure may be insufficient, explaining our results. However, this interpretation is not entirely convincing. First, body representation is highly flexible and can be altered rapidly, as is most spectacularly demonstrated in body illusions [37]. Second, body-size adaptation aftereffects seem to last relatively long, as illustrated by the detection of aftereffects 18 min after a 5 min adaptation period, but they also somewhat tend to fade with time [10]. If mirror exposure was sustained, the perceptual aftereffects should further diminish with time, as the participants' own reflection might cause a deadaptation, progressively nullifying the initial aftereffects. Third, the procedure we used is more controlled than everyday life exposure to bodies. The lack of effect in such laboratory settings is surprising if one expects effects to arise from the unsystematic media and mirror exposure of everyday life, detectable even using single-item scales [1]. Given that we could not detect any self-specific effect in a laboratory study with a relatively large sample (*N* = 196) using psychophysical protocols and during a short duration, it would be surprising that adaptation could explain any substantial misperception of body shape after uncontrolled media and mirror exposure in everyday life.

We chose a short exposure duration as an arbitrary compromise between the fading of the adaptation aftereffect and the time necessary for body image updating, given that these phenomena, along with the number and duration of exposure sessions required for body image updating, have not yet been specified [13]. However, and although unlikely, it remains possible that these effects only begin to appear after several exposure sessions, even in a short-term laboratory study. To test the idea that several exposure sessions are essential to self-specific perceptual effects, future studies could modify our protocol to include multiple cycles of adaptation and mirror exposure. One could hypothesize that exposure sessions allow for a gradual internalization of the distorted mirror reflection into participants' long-term body representation, whereas each session individually would not lead to detectable aftereffects.

In the absence of studies on the effects of repeated and distributed exposure in particular, one might doubt that adaptation aftereffects can be internalized in perceptual body image. Then, how could we account for the gathered evidence around body-size adaptation effects [4,9,13]? Denying that body-size adaptation is the root of body image distortion does not imply denying the existence of adaptation aftereffects on pictures of bodies. As we have argued, the results of most studies, even those focusing on participants' own body representation, can be explained by adaptation aftereffects on visually presented bodies. After adaptation to low-BMI bodies, participants would perceive test stimuli as larger, explaining the difference in normality and comparison judgements, which is unchanged by the body of reference to which they are compared [16]. An adaptation effect that is limited to seen stimuli and does not influence body image would also explain the absence of adaptation aftereffects on body dissatisfaction [43] (but see also [44]) or tactile distance estimation [18].

Instead, sceptics of the internalization hypothesis face a challenge that resides in explaining the effects of the ‘visual diet' on body representation [45]: how can perceptual body image be influenced by mere exposure to extreme-sized bodies if not by visual adaptation? Recent studies investigated body image disturbance using procedures strikingly similar to the body-size adaptation paradigm, but rooted in a social comparison theory approach [14,46–48]. According to social comparison theory, people have an automatic tendency to compare with others [49]. Women who compare themselves to idealized thin media pictures experience increased body image disturbance [47]. In this framework, people may overestimate the size of their own body because they have compared themselves with thinner people, who are overrepresented in the media [1]. The association between exposure to thin bodies, social comparison, and body image distortion has been established in several meta-analyses. Exposure to thin bodies decreases body satisfaction [2] even when participants are not explicitly asked to compare with the stimuli [3] (but see also [48]). In everyday life, people who engage in more social comparison also tend to be more dissatisfied with their body [50].

However, both the operationalization of ‘thin-ideal exposure' and the measure of body image distortion vary greatly in these studies, influencing their results [3,50]. Importantly, studies inspired by social comparison theory tend to focus on attitudinal body image rather than perceptual body image [51] or, more specifically, on body dissatisfaction rather than body shape misperception. For example, Moreno-Domínguez *et al*. [47] measured the effect of exposure to photographs of lower BMI (versus larger BMI) bodies extracted from real magazines. Their measures included a composite body image construct assessed with scales pertaining to their attractivity, but also to their perceived body shape. Exposure to lower BMI (versus higher BMI) bodies affected this body image measure in line with classic aftereffects. Yet, its composite nature makes it unclear whether participants' perception of their body shape had been modified or whether exposure affected participants' body satisfaction.

Future studies should further combine the body-size adaptation and social comparison approaches to study body shape misperception in relation with thin-ideal exposure. In particular, if the effects of adaptation are limited to the perception of bodies in the participants' environment without directly affecting their body image, this does not mean that it cannot have indirect effects. For example, visual normalization theory [52] proposes that weight status assessment can be influenced by the effects of exposure to bodies. Moreover, exposure to large bodies could increase the body size at which someone is categorized as overweight [53]. In this way, adaptation could widen the gap between people's perceived body (which remains stable) and their ideal body (which is represented as ever thinner). This gap between the perceived and ideal body is crucial in the attitudinal component of body image [54] and is associated with young women's body dissatisfaction [55]. These interpretations, although speculative, illustrate how visual normalization and social comparison could inspire new hypotheses about modulators of the effect of media exposure, such as awareness of the comparison situation [48,50], cognitive load [46] or initial body dissatisfaction [14,56]. But these studies ought to make sure that comparison effects influence participants' internal body image and not just test stimuli, beyond non-specific adaptation aftereffects. Adaptation effects can influence the perception of stimuli within minutes, as demonstrated by the large effect sizes we obtained after only 2 min of exposure. This is why, although the internalization hypothesis struggles to explain self-specific misperception, we fully share the recommendation of Brooks *et al*. [13] to beware of adaptation aftereffects on test stimuli while conducting experiments on perceptual body image.

## 5. Conclusion

In conclusion, mirror exposure following adaptation to a high-BMI body did not produce any self-specific perceptual effect. This finding contradicts the internalization hypothesis, which could have explained the emergence of media-related distortions of perceptual body image. Although the aftereffects of body-size adaptation appear to be limited to the perception of seen stimuli, other processes, such as social comparison or visual normalization, might explain the association between exposure to low-BMI bodies in the media and body image distortions.

## Data Availability

The datasets supporting this article have been uploaded as part of the electronic supplementary material [57].
